# Army Medical Department

**Published:** 1861-01

**Authors:** 


ARMY MEDICAL DEPARTMENT.—The Examining Board of Army Surgeons, composed of Drs. C. A. Finley, C. S. Tripler, N. S. Jarvis, and C. H. Smith, Recorder, met in Baltimore on the twentieth of September, and found the following gentlemen qualified for the post of Assistant Surgeons in the United States Army. Their names are given in the order of their relative merit: Dr. Campbell Shorb, Dr. A. F. Mechim, Dr. Clinton Wagner, all of Maryland; Dr. D. P. Ramseur, of North Carolina, and Dr. W. F. Cormick, of Virginia.

